# Functions of lactate in the brain of rat with intracerebral hemorrhage evaluated with MRI/MRS and in vitro approaches

**DOI:** 10.1111/cns.13399

**Published:** 2020-06-02

**Authors:** Yue Liu, Shusheng Yang, Erli Cai, Li Lin, Peng Zeng, Binbin Nie, Fuqiang Xu, Qing Tian, Jie Wang

**Affiliations:** ^1^ State Key Laboratory of Magnetic Resonance and Atomic and Molecular Physics Key Laboratory of Magnetic Resonance in Biological Systems Wuhan Center for Magnetic Resonance Wuhan Institute of Physics and Mathematics Innovation Academy for Precision Measurement Science and Technology Chinese Academy of Sciences Wuhan China; ^2^ Department of Pathology and Pathophysiology School of Basic Medicine Institute for Brain Research Huazhong University of Science and Technology Wuhan China; ^3^ Cell Molecular Biology Laboratory of Basic Medical College Hubei University of Chinese Medicine Wuhan China; ^4^ Key Laboratory of Nuclear Radiation and Nuclear Energy Technology Institute of High Energy Physics Chinese Academy of Sciences Beijing China; ^5^ Wuhan National Laboratory for Optoelectronics Huazhong University of Science and Technology Wuhan China; ^6^ Center for Excellence in Brain Science and Intelligent Technology Chinese Academy of Sciences Shanghai China; ^7^ University of Chinese Academy of Sciences Beijing China; ^8^ Hebei Provincial Key Laboratory of Basic Medicine for Diabetes 2nd Hospital of Shijiazhuang Shijiazhuang China

**Keywords:** emodin, hemorrhages, in vivo MRS, lactate, microglia

## Abstract

**Introduction:**

Lactate accumulation in the brain is caused by the anaerobic metabolism induced by ischemic damages, which always accompanies intracerebral hemorrhages (ICH). Our former findings showed that microglia's movement was always directly toward hemorrhagic center with the highest lactate concentration, and penumbra area has the largest density of compactly arrayed microglia. However, the relationship between microglia and lactate concentration has not been well documented.

**Methods:**

Cerebral hemorrhage model was successfully achieved by injecting collagenase VII (causing stabile localized bleeding) in CPu (striatum) of SD rats. Emodin was used as a potential therapeutic for ICH. The function of the lactate was examined with in vitro culture studies. Then, the effect of lactate on the proliferation, cell survival, migration, and phagocytosis property of microglia was investigated by in vitro culture studies.

**Results:**

Lactate accumulation was observed with in vivo MRS method, and its concentration was monitored during the recovery of ICH and treatment of emodin. Lactate concentration significantly increased in the core and penumbra regions of hemorrhagic foci, and it decreased after the treatment of emodin. The in vitro culture study was verified that lactate was beneficial for the proliferation, cell survival, migration, and phagocytosis property of the microglia.

**Conclusion:**

Results from in vitro verification study, investigations from the recovery of ICH, and treatment of emodin verify that lactate plays an important role during the recovery of ICH. This could provide a novel therapeutic approach for ICH.

## 1 INTRODUCTION

Intracerebral hemorrhage (ICH) accounts for 8%‐15% of all strokes in the high‐income countries^1^ while the current research efforts only have limited effects. Multiple complicated factors including the initial hematoma volume, hematoma expansion during the acute phase, location of the hematoma, extent of brain edema, age, and neurological status on admission all influence the clinical outcomes.^2^ However, the brain energy metabolism and CNS immune system are undoubtedly important factors in brain function restoration after ICH.^3^, ^4^

Normally, glucose is the major energy source for the brain under normal physiological conditions. Many studies also indicate that lactate is an efficient substrate for the brain.^5^, ^6^ In the brain, lactate is formed predominantly in astrocytes from glucose or glycogen and released *via* the monocarboxylate transporter 4 (MCT4) and monocarboxylate transporter 1 (MCT1), and transported by MCT2 into neurons, where it is converted to pyruvate that is subsequently metabolized through oxidative phosphorylation. In a study using ^13^C‐L‐lactate and magnetic resonance spectroscopy (MRS) on young, healthy volunteers, it was suggested that at normal physiological peripheral levels L‐lactate may contribute 10% to brain metabolism, but this can rise to 60% at supraphysiologic levels.^7^ The transference of lactate to neuron, for example, the astrocyte‐neuron lactate transfer shuttle system, satisfies neuronal energy needs, but also modulates the signals on neuronal functions, including excitability, plasticity, and memory consolidation.^8^

When the brain cannot get enough oxygen or is unable to deal with oxygen sufficiently or fast enough, the concentration of lactate increases substantially. Thus, lactate has generally been associated with the anaerobic metabolism induced by ischemic damages, and the elevated lactate is regarded as the marker of cell hypoxia and poor neurological outcomes. Lactate is both created and consumed in aerobic conditions, and serves as a link between glycolytic and oxidative metabolism.^9^ Apart from the basic roles during brain metabolism, lactate also plays a major role in CBF regulation^10^ and vascular modulation.^11^ Furthermore, lactate accumulation is also linked to ICH. In recent studies, it also contributed to the potentiating of angiogenesis and neurogenesis.^12^ However, the measurement of lactate concentration in these studies was inappropriate due to its dramatic increase after euthanasia of the animal test subject. Therefore, further studies are needed to explore the roles of elevated lactate after brain damage, such as ICH.

To understand the molecular and cellular mechanisms underlying early brain damage after ICH, we injected 0.4 U collagenase VII into the left caudate putamen (CPu) of rats and successfully duplicated an ICH rat model evaluated by behavior tests and brain magnetic resonance imaging (MRI). Through in vivo magnetic resonance spectrum (MRS) tests, the elevated lactate was found in the hematoma regions of ICH rats. In the combination with other techniques, such as the study of cultures, animal behaviors, immunohistochemistry, and emodin treatment, the function of lactate accumulation in ICH was investigated in this study. This study could provide a novel therapeutic approach in ICH treating.

## 2 MATERIALS AND METHODS

### 2.1 Animals

The animal protocol was approved by the Animal Care and Use Committee of Wuhan Institute of Physics and Mathematics, Chinese Academy of Sciences. Three‐month‐old male Sprague Dawley rats (250 ± 10 g) were purchased from the Center for Disease Control and Prevention in Hubei province, China. They were housed in a 12‐hour/12‐hour light‐dark cycle, temperature and moisture autocontrolled specific pathogen‐free animals (SPF) animal room. Food and water were available ad libitum. In order to minimize the stress and accustom the animals to human interaction, all animals were handled daily for one week until the day of the experimental.

### 2.2 Antibodies and chemicals

Collagenase VII and 4′,6‐diamidino‐2‐phenylindole (DAPI) were from Sigma. Collagenase VII was dissolved in physiological saline solution at concentration of 25 U/μL and stored at −20°C. Before use, collagenase VII was diluted to 0.2 U/μL in 0.9% NaCl. All the antibodies used in this study are listed in Table S1. Prussian blue staining kits were from Leagene; Histostain TM‐SP kits were from ZEMED; and diaminobenzidine (DAB) was from ZSGB‐Bio. Emodin was obtained from Shanghai Base Industry and dissolved in DD water (0.6%). Lactate was obtained from Sigma.

### 2.3 Experimental design

The current study includes two parts. In the first part, the lactate accumulation in the ICH animal model was verified, as was the therapeutic efficiency of the potential drug emodin to cure the ICH. The functional role of lactate accumulation in brain function was also proposed in this part (Figure S1). The second part was concerned with verifying the functional role of lactate accumulation in brain function with traditional culture study.

### 2.4 Surgery and emodin administration

To generate the ICH animal model, the traditional method of intracerebral collagenase injection was applied to the animals.^13^ The stereotactic injections were completed in a stereotactic injection system. The detailed steps of ICH animal model generation were described in our pervious study^14^; thus, only brief steps were provided here. 2 μL (0.4 U) collagenase VII was stereotaxically injected into CPu of the rat brain for the generation of the ICH model, and the vehicle solution 0.9% NaCl (2 μL) was also injected into the same location in sham group. The rate of infusion was controlled in 0.4 μL/min.

To evaluate the functions of lactate in ICH model, a potential drug emodin, which was the major efficient component in the traditional Chinese medicine for ICH—Da‐cheng‐qi decoction, was utilized to treat the upper ICH. The drugs were given once per day one week by intragastric administration, and the saline vehicle was used as the control. The dosage of emodin was converted according to body surface area of human (70 kg) and rat (200 g). The conversion coefficient is 0.018. The dose of 100 mg/kg emodin was used in the following experiments. Then, a series of tests were administered to the treated animals, such as MRI, MRS, animal behaviors, and immunohistochemistry (Figure S1). The number of samples in each group (model, control, emodin‐treated) was 5‐10 rats.

During the experiment period after modeling, the 3rd, 14th, and 28th days were selected for MRI and in vivo MRS studies to investigate the volume of the hemorrhage volume and the tendency of the concentrations of lactate. And the 1st, 3rd, 7th, 14th, and 28th days, animal behavioral studies were applied, including the balance beam^15^ and elevated body swing^16^ tests. The procedures of these two studies were exactly the same as in our previous study^14^; thus, their detailed descriptions are omitted here.

### 2.5 MRI and in vivo MRS studies

All measurements were performed on a Bruker Biospec 70/20 USR small animal MR system (Bruker BioSpin MRI, Ettlingen, Germany) equipped with self‐shielded gradients. A four‐channel phased array surface coil (Bruker BioSpin MRI) was used for both MRI and MRS measurements. In this study, several different animal models were utilized for MRI and in vivo MRS studies, including the ICH model animal, emodin treatment, or control animal groups.

The rat was anesthetized with 4%‐5% isoflurane at first and then transferred to the animal bed in the MRI platform. The isoflurane concentration was lowered to 0.8%‐1.5% to maintain the state of anesthesia during the whole of the MRI and MRS studies. The respiratory rate was monitored with a pressure‐sensitive pad under the abdomen of the animal, and body temperature was maintained using the cycling water heating pad and monitored with a rectal thermometer. The animal was also covered with a piece of cloth to help maintain its body temperature. At the end, a pair of foam ear plugs were utilized to attenuate disturbance from equipment noise. The MRI and MRS experiments then proceeded, and the parameters are described here:

The pulse sequence of fast spin‐echo sequence (Turbo‐RARE) was used to obtain the T2 weighted anatomical images of the animals, field of view (FOV): 20 × 20 mm^3^; echo time (TE): 10 ms; effective echo time: 10 ms, matrix size: 128 × 128; repetition time (TR): 2000 ms; RARE factor: 4; number of averages: 8; spatial resolution: 0.156 × 0.156 × 0.6 mm^3^; and 12 slices without any gap. The acquisition time (Tacq) per image was ~8.5 minutes.

**FIGURE 1:**
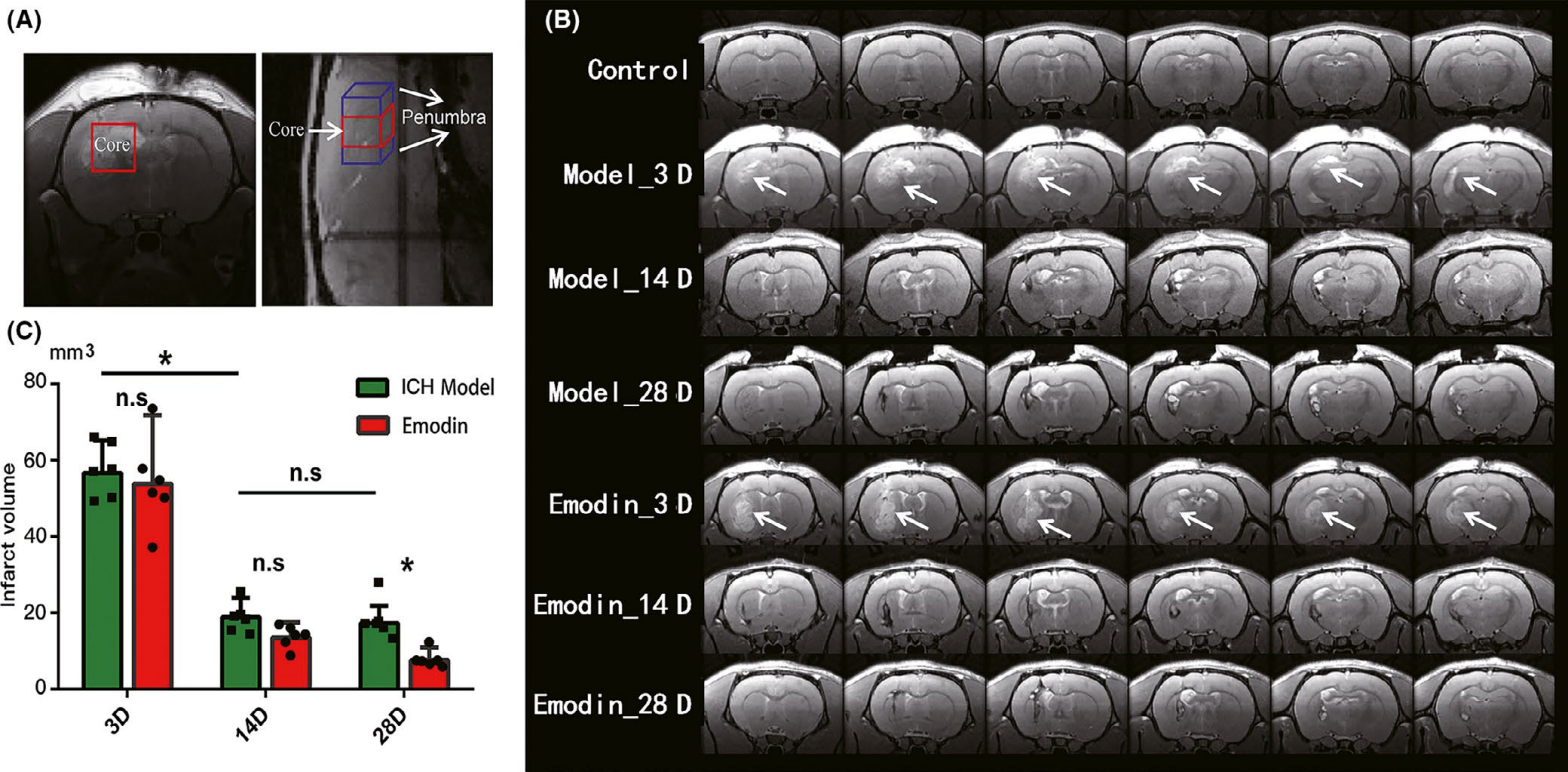
Diagram and volume of intracerebral hemorrhage areas. A, The sketch map of regions being selected for MRS scanning (red cuboid, the core region; blue cuboid, penumbra regions). The dimensions were 3.5 mm (L/R), 3.5 mm (S/I), and 2.4 mm (A/P) in a coronal view. Here, we regard two isometric cuboids around hemorrhagic core region (1/4 overlapping with the core) as the penumbra regions (sagittal slice on the right). B, Coronal scans of model ICH and emodin‐treated rats are shown at time points of 3, 14, and 28 d after injection. The white arrows indicate the highlighted lesion areas of ICH in T2WI images. The figures are from the same rat (each group) in this 28‐d period. And we use nonsurgery rats as control group in MRI and MRS tests to exclude injection‐induced injuries. C. The hemorrhage volume was reconstructed by areas with 20% or above higher signal than contralateral brain areas. And the volume contains dark signals of areas of necroses and cavitations surrounding hemorrhage at 14 or 28 d. *P < .05

For the measurement of the in vivo MRS study, the conventional localized point‐resolved spectroscopy (PRESS) pulse sequence was utilized. The parameters were set as follows: TE: 20 ms; TR: 2500 ms; number of averages: 128; number of dummy scans: 4; spectrum width: 13.34 ppm; and size of FID: 4096. The voxel dimensions were 3.5 mm (L/R), 3.5 mm (S/I), and 2.4 mm (A/P). There were three different voxels selected, the core region of the intracerebral hemorrhage, and two sides of the hemorrhage region with 1/4 region overlapping (Figure 1A). Tacq per spectrum was ~5.5 minutes.

### 2.6 Morphological techniques on brain slices

Rats were anesthetized by over‐dose of isoflurane and transcardially perfused with 100 mL ice‐cold normal saline followed by 500 mL ice‐cold phosphate buffer containing 4% paraformaldehyde. The brain was collected and sunk with 20% and 30% sucrose gradient. Then, the fixed brain was cut into sections (20 μm) with a freezing microtome (Cryostat 1720, Leitz, Wetzler, Germany) for staining—immunohistochemistry and Prussian blue staining.^14^

For immunohistochemistry, the brain sections containing CPu were performed as previously described by our team.^14^ Briefly, the brain sections were incubated for 24 hours at 4°C with primary antibodies Iba‐1 (1:200 Wako). The slices were incubated with Histostain TM‐SP kits and color‐reacted with DAB according to the manufacturer's instructions for 2‐10 minutes. Then for Prussian blue staining, the brain sections were incubated with Prussian blue staining solution and nuclear solid red dye counterstain as previously described.^14^

### 2.7 Effects of lactate on microglial activity in vitro study

Our previous study has shown that the microglia/macrophages presented high morphologic plasticity in the pathogenesis of ICHs accompanied by the accumulation of lactate.^14^ Thus, it was valuable to investigate the relationship of the microglial activity and lactate concentration. Normally, brain extracellular fluid (ECF) lactate concentration is around 1.21 ± 0.06 mmol/L.^17^ The signal intensity of the lactate resonance at 1.3 ppm increased 2‐20 times during the 28‐day recovery period for ICH. In order to investigate the function of lactate accumulation in ICH, 1.21 mmol/L was selected as the base level, and the cell culture was treated with the medium with 2.42, 6.05, 12.10, and 24.20 mmol/L lactate (Sigma), respectively. Microglia cell was used for phagocytosis assay, and the normal BV2 cell was used for analyses of proliferation, cell survival, and migration.

#### 2.7.1 Cell culture

Primary microglia were derived from P1‐P3 (postnatal days 1‐3) rat brains. Briefly, the whole brain was aseptically removed and dissociated after trypsinization (0.25% trypsin, 20 minutes). The whole material was passed through a 100 mm nylon mesh and plated in poly‐L‐lysine–coated T‐75 culture flasks in DMEM (high glucose) supplemented with 15% FBS, 100 units/ml penicillin and 100 μg/mL streptomycin. The mixed glia cultures were maintained at 37°C in a 5% CO_2_ humidified atmosphere. After 7 days, the growth medium was replaced and changed every third day until the 14th day. At the end, microglia were harvested from the mixed glial culture using a rotating shaker at 140 rpm for 6 hours. Cells were collected and plated at a density of 1 × 10^5^ cells per 500 μL medium on poly‐L‐lysine–coated 24‐well plates for future study. Furthermore, the BV2 cells were cultured at 37°C in a 5% CO_2_ humidified atmosphere in DMEM supplemented with 10% FBS, penicillin at 100 units/mL, and streptomycin at 100 μg/mL.

Cell Counting Kit‐8 (CCK8) was used to evaluate the activity of cultured cells with or without lactate treatment. Cells were seeded in 96‐well plates with a volume of 100 μL per well, added various concentrations of lactate medium, respectively, followed by incubation for 24 hours. At the end of incubation, 10 μL of CCK‐8 reagents was added to each well and incubated at 37°C for another 1 hour. The numbers of viable cells were assessed by measurement of absorbance at 450 nm.^18^ The relative assessments of microglial injury were normalized by comparison with control cell at 100% cell survival (CCK‐8).

#### 2.7.2 Microglial migration—in vitro scratch assay

BV2 cells were cultured as confluent monolayers in 6‐well plates and then scratched with a 100 μL pipette tip. The scratched monolayers were washed twice to remove nonadherent cells and media were changed with pure DMEM^19^ as well as DMEM containing various concentrations of lactate. Then, the cells were transferred to the Cell‐IQ for observation and further analysis.

Microglial migration was viewed livelily under Live Cell Kinetic system. Images of multiple positions in each well were captured at the different time points until the wound closed. Using Cell‐IQ (Live Cell Kinetic Imaging & Quantification) analyzer, the wound area was defined in each image by positioning lines in correspondence to the original scratch and the following data were exported. Proliferation of microglia was also observed as above. A Cell Counter protocol was made to measure the ability of microglia to proliferate.

#### 2.7.3 Phagocytosis assay

Cells were cultured in a 96‐well plate at a density of 0.2 × 10^6^ cells per well. The final concentration of FITC‐dextran was 1 mg/mL, and the cells were incubated for 60 m after adherence and washed twice with PBS. For digestion, 50 μL trypsin was added for 2 m. 200 μL FACS buffer (0.05% BSA in PBS) was added, and the cells were determined by a flow cytometer (FACSAriaIII). For immunofluorescence staining, free‐floating slices were incubated at 4°C overnight with anti‐CD68 and anti‐Iba‐1. The immunoreactivity of anti‐Iba‐1 was probed using Alexa Fluor^®^ 488 anti‐goat IgG (H + L). The immunoreactivity of anti‐CD68 was probed using Alexa Fluor^®^ 546 anti‐mouse IgG (H + L). Images were observed using a laser scanning confocal microscope (Zeiss LSM 780, Germany).

### 2.8 Calculation method and statistical analysis

Normally, the concentrations of creatine and water were assumed to be stable,^20^ especially for creatine is indeed stable across various conditions including pain.^21^, ^22^ Thus, creatine was always used as the internal concentration reference, and the relative concentrations of other metabolites were provided with the ratio of their concentration to the inner reference (creatine). Here, the concentration of the metabolites in the in vivo MRS data was calculated with ratio of areas between the related peak and the creatine peak. Furthermore, the area of lactate signal was seriously overlapped with macromolecule at 1.3 ppm,^23^ it was very hard to estimate the initial concentration. However, the increase of the relative concentration of lactate was easily calculated with subtraction of the peak areas between the treated model and control model.

Data were analyzed by using GraphPad Prism (GraphPad Software, Inc). Normally, independent Student's *t* test (2‐tailed, *P < .05, **P < .01, ***P < .001) method was used in comparison of every two groups. For the data not in accordance with normal distribution, 2‐tailed nonparametric test (Mann‐Whitney test) was used instead (*P < .05, **P < .01, ***P < .001). Results were presented in average ± standard deviation (Ave. ± SD).

## 3 RESULTS

### 3.1 Hemorrhagic areas in the hemorrhage animal model

Similar with our former study, 0.4 U collagenase VII was injected into the left CPu of SD rats (n = 6) for the construction of hemorrhage animal model. The hemorrhagic foci were continuously monitored using MRI method in the 3rd, 14th, and 28th days after the surgery. The MRI results in Figure 1B were collected (one rat from each group for the duration as an example). The hemorrhagic areas were successively observed 3 days after the surgery, and the area was gradually decreased in the following days. Statistical analyses of the hemorrhagic areas were collected in Figure 1C. The volumes of hemorrhage of ICH model group were 56.7 ± 8.4, 18.9 ± 5, and 17.3 ± 4.6 (mm^3^) (3rd, 14th, and 28th). The area was significantly decreased after 14 days (*P = .016) and kept stable (n.s *P* = .805). The metabolites in the brain lesion area were measured with in vivo MRS method synchronously.

### 3.2 Lactate concentrations in the core and penumbra regions of hemorrhagic foci

**FIGURE 2:**
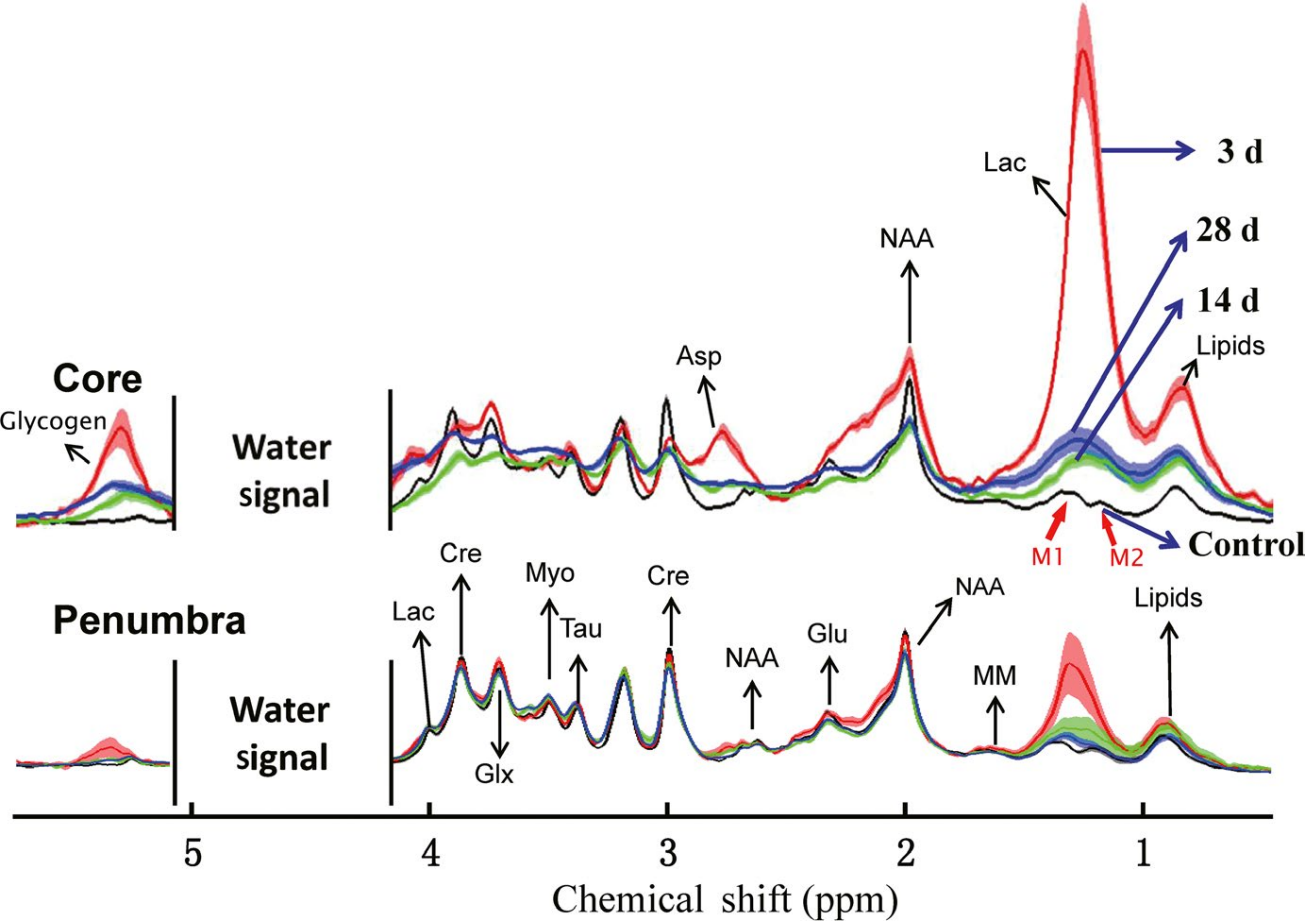
In vivo ^1^H MRS results show the lactate concentration changes in ICH. The spectroscopies of core and penumbra regions at 3 time points. Discordant water signal was deleted to show other metabolites obviously. Lactate signal in the higher magnetic field (~1.3 ppm, blue arrows) was used for qualitative and quantitative analysis

The metabolites in the core and penumbra regions of hemorrhagic areas were measured with in vivo MRS approach as described above. The identified metabolites in the NMR spectra were labeled in Figure 2 (Asp, aspartic acid; Cre, creatine; Glyco, glycogen; Glu, glutamic acid; Glx, glutamate and glutamine; Lac, lactate; lipids; MM, macromolecule; Myo, myo‐inositol; NAA, N‐acetyl aspartate; Tau, taurine). The lactate concentration was significantly increased 3 days after the surgery (*P* < .0001, 11 times higher vs. control group). Then, it was significantly decreased but still higher (2‐4 times in relevant value) than the control group 14 days and 28 days after surgery (*P* < .001). Furthermore, the other energy metabolites were also significantly increased in the core region, such as Glyco,^24^ aspartate (Asp),^25^ and lipids, similar to the results of microdialysis method.^26^ Both Glyco and Asp were almost undetectable under the normal condition in our 7.0T MRI machine (black spectrum in Figure 2), and it was significantly increased in both treatment models. However, they were very hard to quantify due to the influence of water suppression (for Glyco) and baseline disturbance after hemorrhage (for Asp). In the penumbra region, the patterns of the NMR spectra were almost similar to the control group, but the lactate's concentration was also significantly higher 3 days after the surgery. Comparing with the other metabolites, lactate was the major altered chemical in the whole hemorrhagic area.

### 3.3 Treatments of emodin on the hemorrhages

#### 3.3.1 Normal dynamic changes of the rats' weight

At the beginning, the weights of the rats significantly decreased, probably due to the intake of food and water being substantially reduced, especially 3 days after ICH (Δweight: Con 5.20 ± 7.86 g, model −16.75 ± 8.23 g, emodin −22.20 ± 15.59 g). And then with the increase of food and water intake, the weight of every group gradually increased and there were no significant differences among the groups after 28 days (Figure S2).

#### 3.3.2 Athletic ability of ICH model rats

**FIGURE 3:**
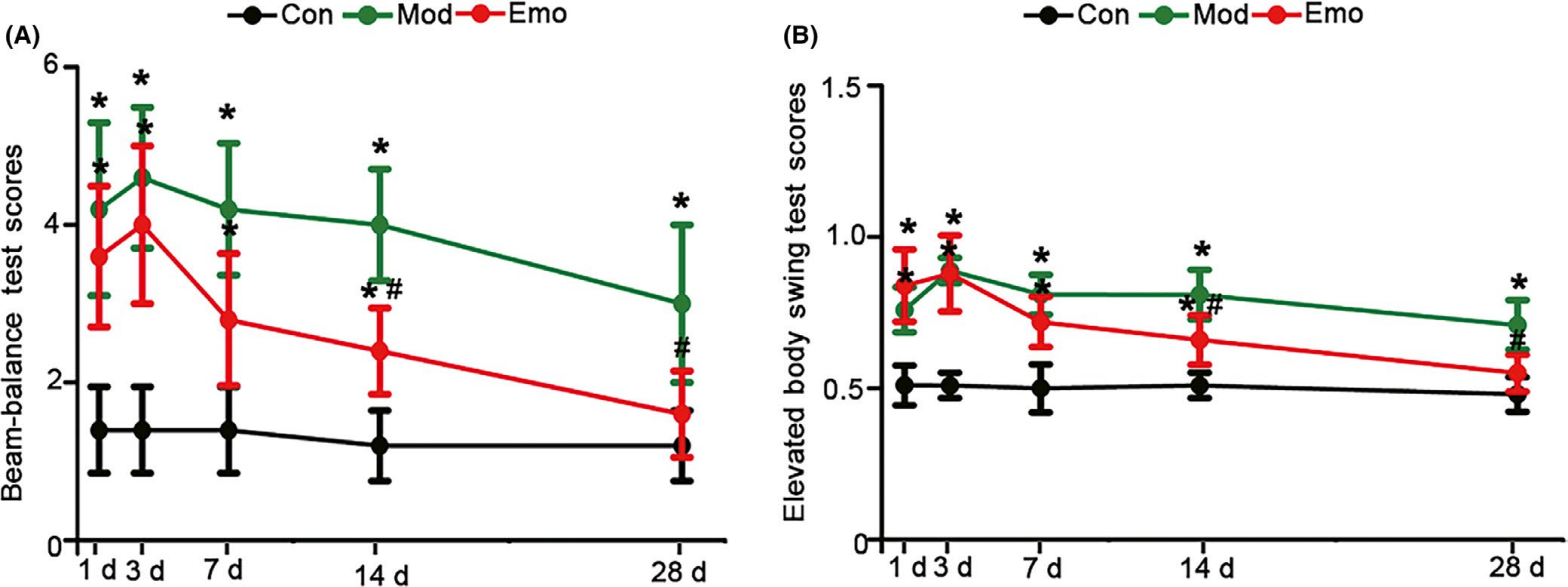
Emodin could reverse the athletic ability of ICH. The balance beam test A and the elevated body swing test B were used to assess the athletic ability of the control rats, model rats and the ICH rats being treated with emodin after the injection of collagenase VII for 1 d, 3 d, 7 d and 14 d and 28 d. The data were expressed as means ± SD (n = 5). *P < .05 vs Con, #*P* < .05 vs Mod

There were obvious abnormal behaviors in the ICH model rats. In the balance beam test, the model rats showed a decline in action coordination, and they were prone to falling from the right‐hand side. This indicated that the model rats had brain damage on their left sides and were unable to maintain the balance. This lasted for 28 days. The frequency of their falling from the right side reduced obviously as the model rats were treated with 100 mg/kg dose of emodin after a 14‐day period. There was no difference between the treated group and the control group when the rats were treated with emodin for 28 days (Figure 3). Meanwhile, the elevated body swing test was also used in our study and the bodies of the model rats turned to the left side, which was the bleeding side. Although abnormal movement improved to some degree on the 28th day compared with that on the 3rd day after collagenase VII, the station of those groups treated with emodin recovered earlier and the rats returned to normal after being treated for 14 days (Figure 3).

#### 3.3.3 Areas of hemorrhages in both models

After the ICH animals were treated with emodin, MRI and MRS methods were also applied over three different time points. Areas of hemorrhages were measured (Figure 1). With the treatment of emodin, hemorrhage areas were 53.80 ± 17.94 mm^3^ and there was no significant difference in 3 days after the surgery in ICH rats. Similar to the ICH model, the total hemorrhage areas were dramatically decreased 14 days after surgery (13.50 ± 3.99 mm^3^) and even continued to reduce on the 28th day (7.65 ± 3.34 mm^3^). There was a decreasing tendency after 14 days and a significant difference observed 28 days after the treatment of emodin (n.s *P* = .200, *P = .014).

#### 3.3.4 Lactate concentrations in the hemorrhages related areas

**FIGURE 4:**
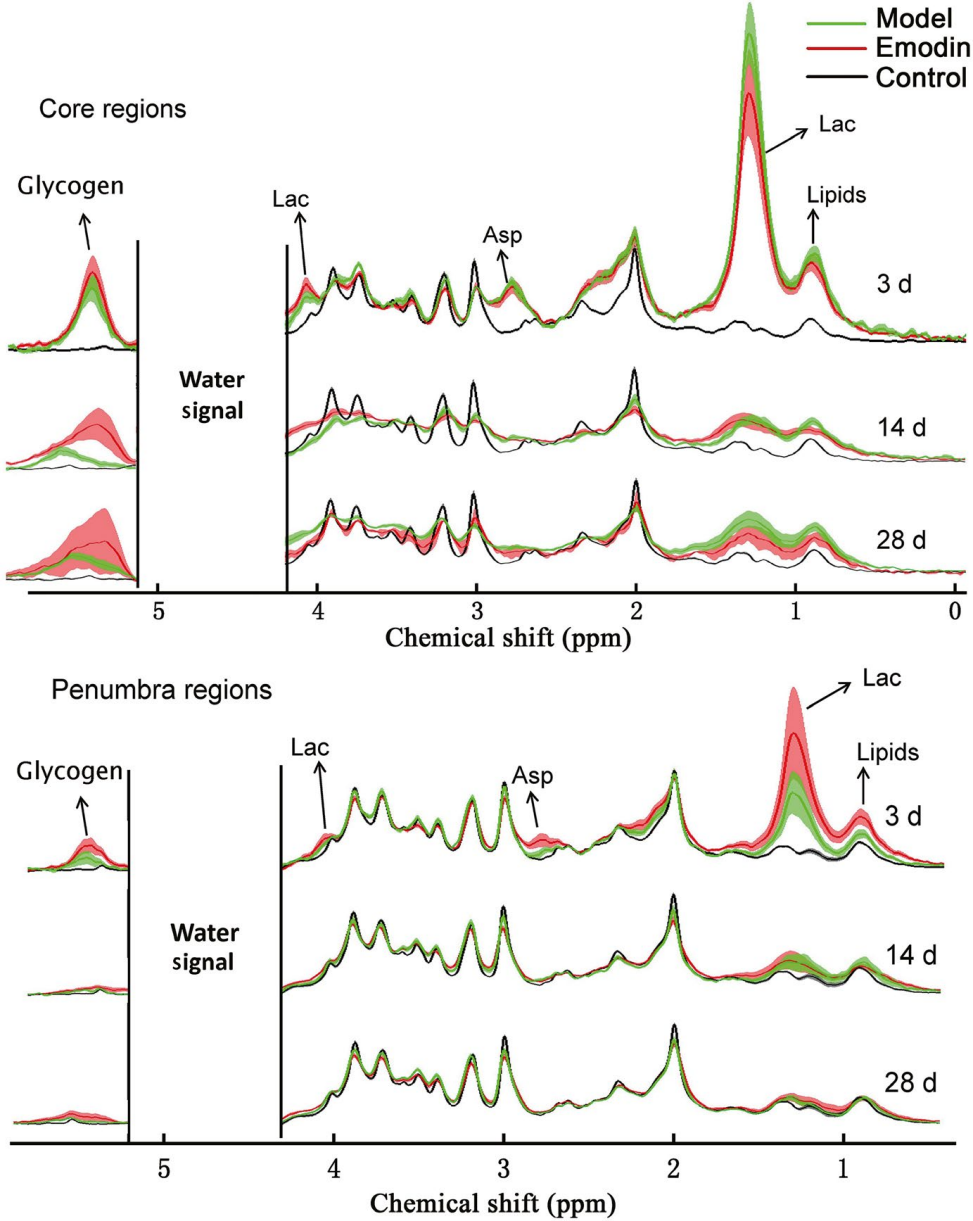
The comparison of MRS in model and emodin‐treated groups. Discordant water signal was deleted to show other metabolites obviously. Asp, aspartic Acid; Glyco, glycogen; Lac, lactate

After treatment of emodin, the dynamic changes of the metabolites in the core and penumbra regions were measured 3, 14, and 28 days after the surgery (Figure 4). Similar to the ICH model, the shape of the MRS spectrum was seriously destroyed in the core region. Much higher lactate concentration (increased 11.43 times compared to Con) was observed in the core region during the acute stage, even with the treatment of emodin. It then gradually decreased in the following days. There were no significant differences observed to the lactate concentration in the core region during different time points. For the penumbra region, it was very interesting that the lactate concentration increased after 3 days of emodin treatment in the ICH rats (6.44 times vs Con, and 2.08 times vs ICH model). It then dropped down to around normal level 14 and 28 days after the surgery. Similar to the ICH model, metabolites of Glc, Asp, and lipids were also different 3 days after surgery in both regions. However, the other metabolites in the penumbra region were gradually recovered after 14 days, and were stable after 28 days.

#### 3.3.5 Pathological changes in brain tissue

**FIGURE 5:**
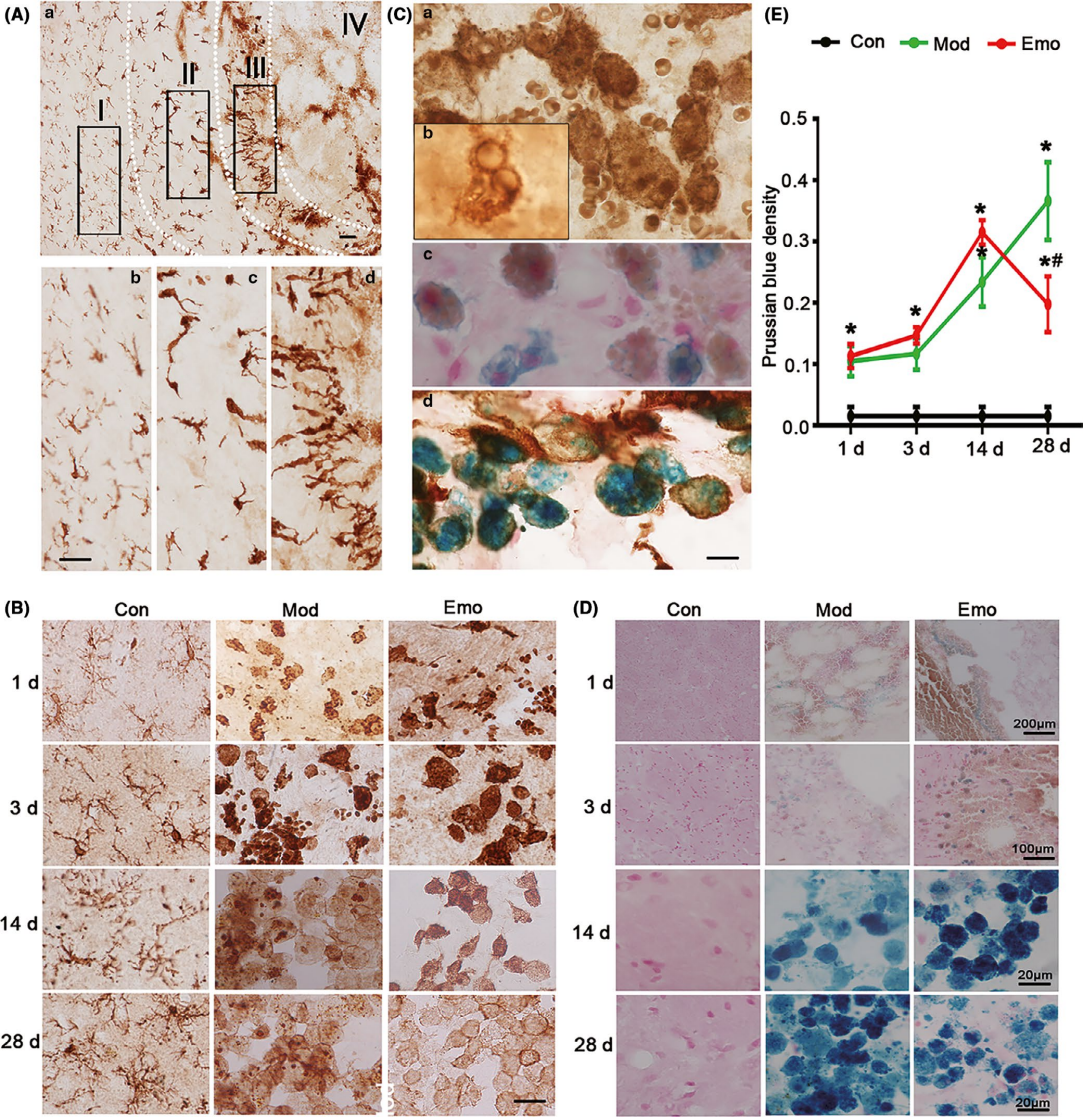
Emodin could improve the morphologic change of microglia/macrophages and reverse the pathological changes of iron deposition in the hemorrhagic CPu of ICH rats. A, After the behavior test, the brain slices (20 μm) in the hemorrhagic CPu were used to assess the morphologic alterations of microglia/macrophages by immunohistochemistry staining and pathological changes of iron deposition by Prussian blue staining. By immunohistochemistry staining on brain slices, Iba‐1 (marker of microglia/macrophage)–positive cells shown different morphological distribution in different areas when collagenase VII was injected in CPu for 3 d. B, Furthermore, Iba‐1–positive cells presented pathological changes in different time and emodin could ameliorate the changes. C, Microglia/macrophages could phagocytize red blood cells which also were positive by Prussian blue staining. D, And then Prussian blue staining was used to value the iron deposition of hemorrhagic lessons. The average degree of density means of Prussian blue stain was detected by IPP software when the injection of 1 d, 3 d, 7 d, 14 d and 28 d. The square area (S) and average degree of mean optical density (MOD, deducting background absorbance) were counted with IPP software and then calculated the density (D, D = S×MOD) of every colored area. E, The total colored square area (Σ*S*) and total Prussian blue density (Σ*D*) of every brain section were counted and then the average MOD of total colored area of every brain section (MOD = Σ*D*/Σ*S*) was also counted. The data were expressed as means ± SD (n = 10). *P < .05 vs Con, #*P* < .05 vs Mod

To verify the cells around the hemorrhagic foci, the brain slices of ICH rats were stained with antibodies specific to Iba‐1 (marker of microglia/macrophages) by immunohistochemistry staining at the days of 1, 3, 14, and 28 after operation (Figure 5). The microglia/macrophages were observed increasing (~2700/mm^2^ in ICH area vs ~250/mm^2^ in contralateral area) and accumulating in the collagenase VII‐injected CPu with the pathological change into ameboid type (Figure 5A). Emodin could accelerate the activation of microglia and the pathological process of recovery in ICH rats (Figure 5B).

Blood composition, including red blood cells, would enter into the brain tissue through the destruction of the blood brain barrier (BBB) after ICH. The local brain environment would change radically, such as inflammatory markers released and gathered. Brain neurons would be destroyed and release abnormal substances. Among these intracellular substances being released to the local brain tissue, iron was important and had far‐reaching influence on the pathological change of ICH. In order to observe and evaluate the iron deposition that entered into the brain, Prussian blue staining was used to analyze the average degree of mean optical density (MOD) (Figure 5C‐E). The results of Prussian blue staining indicated the colored degree of model rats rose gradually after collagenase VII injection from 3 days to 28 days, which illustrated the pathologic change of iron deposition in the model rats aggravated progressively (Figure 5D). Although iron deposition existed in the rats treated with emodin for 1 day to 14 days and reached to peak value on 14 days, average degree of mean optical density (MOD) of Prussian blue iron stain reversed obviously when rats were treated with emodin for 28 days (emodin vs Mod, *, #*P* < .05, Figure 5E). These data indicated that emodin could alleviate the pathologic change of iron deposition of ICH model rats.

From Figure 5C,D, iron ions were phagocytized by activated microglia. Therefore, both of these two figures also showed microglia activation can accelerate recovery after cerebral hemorrhage.

### 3.4 Lactic acid enhanced microglial abilities of migration, proliferation, and phagocytosis

**FIGURE 6:**
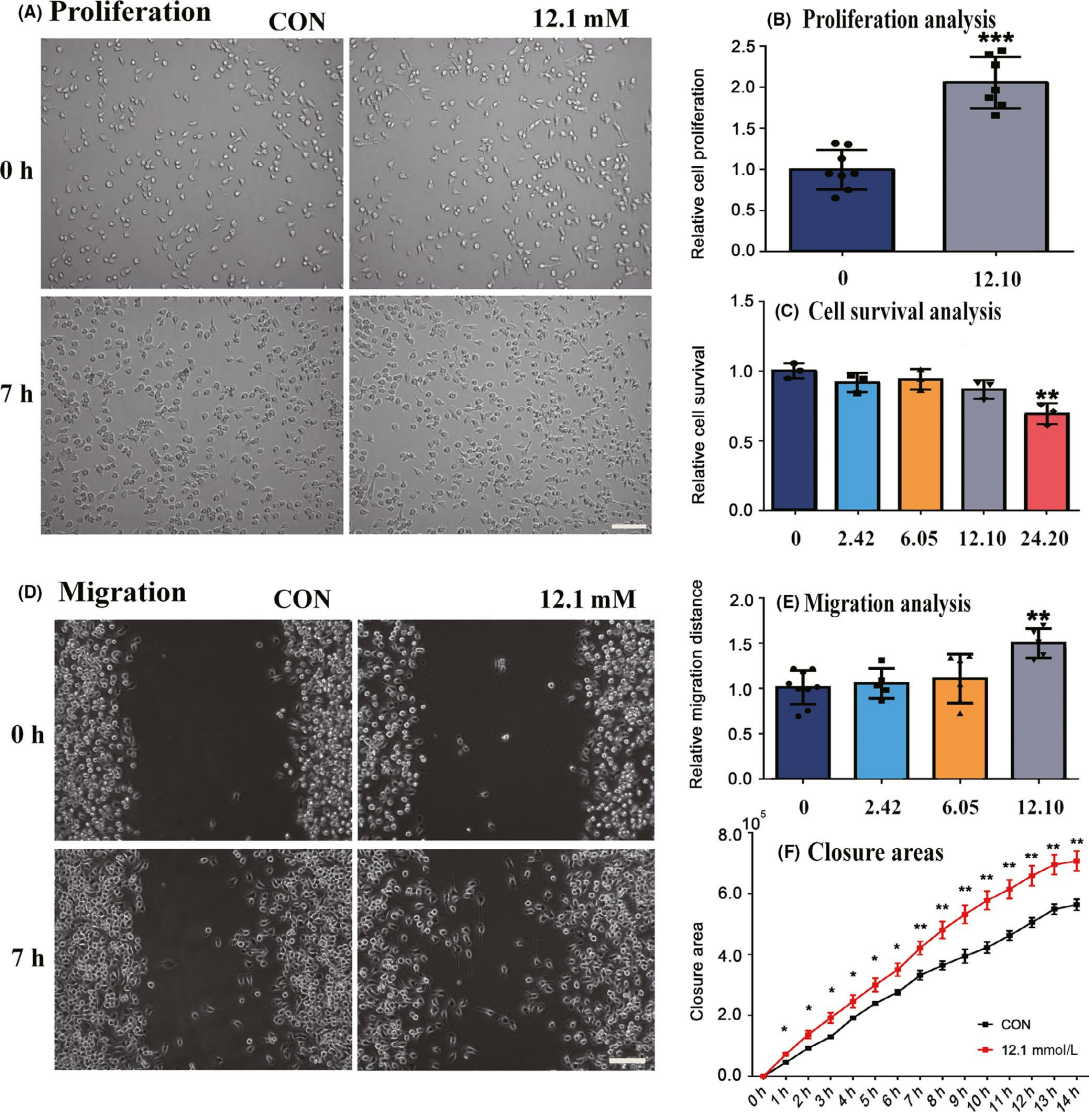
Elevated level of lactic acid induced migration and proliferation of microglia. A, In vitro scratch assay was performed and the images of migration were captured at 0 h and 7 h after scratching with Live Cell Kinetic system. B, By cell counting, the relative proliferation was quantified as defined above in triplicates. C, The effects of lactate on microglial toxicity. D, The observation for cell proliferation was carried out by imaging captured at 0 h and 7 h. E‐F, The migration distance and the closure area were quantified by Cell‐IQ analyzer from three independent experiments. Data were presented as means ± SD *P, .1, **P, .05, ***P, .01 versus lactate‐treated cells

Microglia activation and proliferation may occur in almost any single pathology affecting the CNS.^27^ When the lesions occur, nearby microglia were activated from ground to excited. Activated microglia exhibit high capacity of proliferation and migration.^28^ As extremely high concentration of lactate in hemorrhage areas, we wonder whether there is a relationship between lactate concentration and microglia or whether the activation of microglia is caused by excess lactate. To explore the effects of lactate concentration on microglial proliferation, we counted the same batch of BV2 cells under different lactate concentrations. According to in vivo MRS results, we choose 2.42 mmol/L (normal), 12.10 mmol/L (penumbra), and 24.20 mmol/L (core) as the concentration gradient in vitro experiments. In the treated group (12.10 mmol/L), the numbers of BV2 were obviously increased after 7 hours (Figure 6A,B), which indicated the proliferation of microglia. Lactate (2.42‐12.10 mmol/L) had no cytotoxicity on microglia; nevertheless, the cell viability was significantly reduced when the lactic concentration reached 24.20 mmol/L (Figure 6C). All these data revealed that the proliferation rate of microglia increased when the concentration of lactate reached 12.10 mmol/L. Thus, this concentration was selected as the highest concentration for the migration rate analysis.

To investigate the effects of lactate on the migration of microglia, we used the in vitro wound scratch assay as well as the Cell‐IQ (Live Cell Kinetic Imaging & Quantification) for long‐time incubation and imaging. We observed an increased migration rate of microglia cultured with 12.10 mmol/L lactate (Figure 6D,E), whereas microglial migration in the other two groups (2.42 mmol/L and 6.05 mmol/L) had no statistical significance in the first 7 hours (Figure 6E). We also calculated the total closure area‐time curve and found that closure of the wound was more rapid in the experimental group (12.10 mmol/L) (Figure 6F).

**FIGURE 7:**
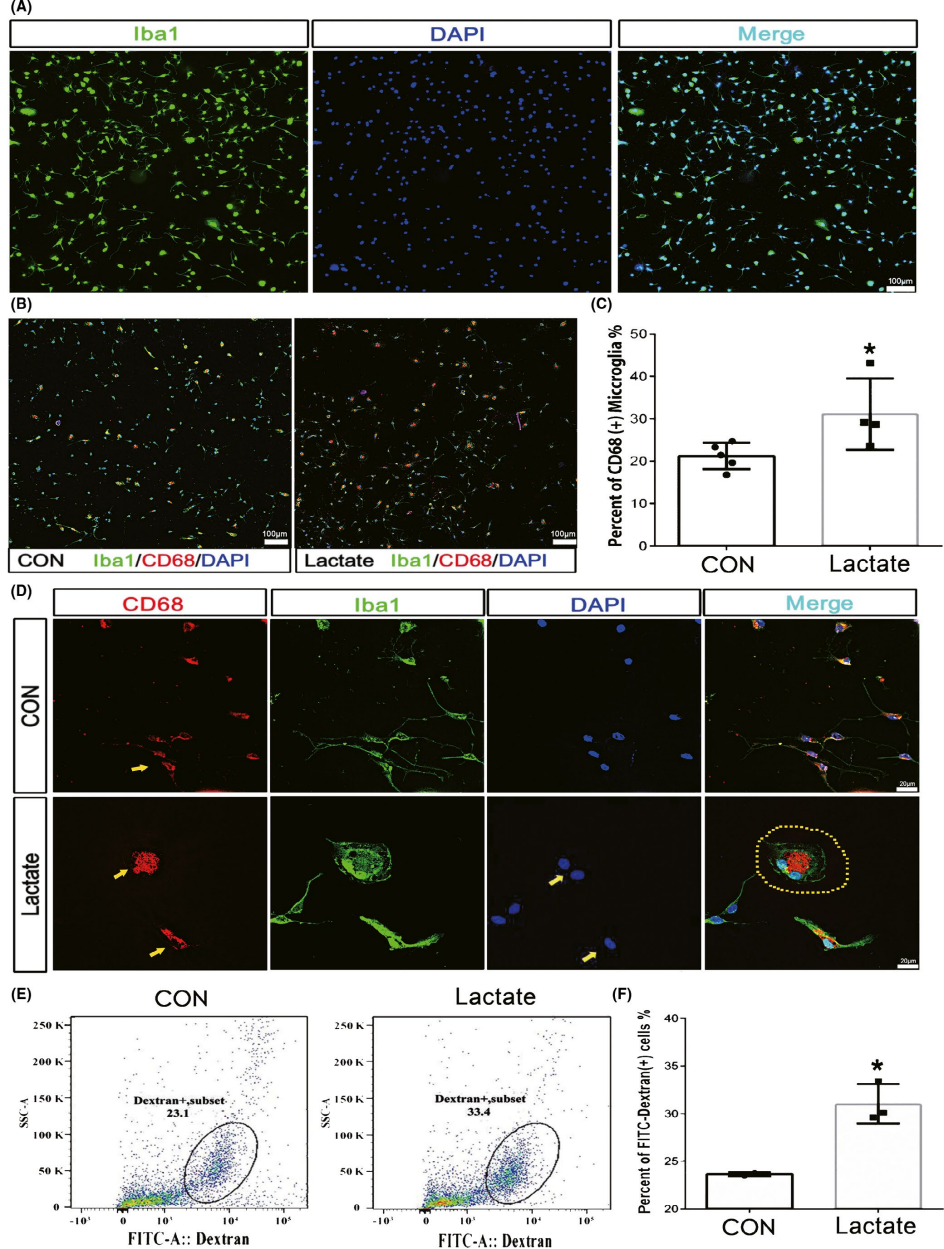
Elevated level of lactic acid enhanced phagocytosis of microglia. The purity of primary microglia reached 99% (A). CD68^+^/Iba1^+^ cells and Iba1^+^ cells were separately counted with low magnification (10×) under fluorescence microscopy (B) and the percentage of CD68^+^ microglia showed an enhancement of microglial phagocytosis (C). Microglia exhibited an activated phenotype and enhanced phagocytosis induced by lactate with high magnification (60×) under fluorescence microscopy (D). The percentage of microglia engulfing FITC‐dextran increased (E, F). The upper bar = 100 μm, the middle bars = 20 μm, and the lower bar = 100 μm. Data were presented as mean ± SD, *P < .05 vs control microglia

We further carried out immunofluorescence staining of primary microglia for CD68, a lysosomal activity marker, and Iba‐1. The purity of primary cultured cells was first assessed to guarantee the reliability of our experiment and the result showed highly purified microglia (Figure 7A). Our results suggested a 10% significant higher proportion of CD68 positive microglia (to total microglia) when the concentration of lactate reached 12.10 mmol/L (Figure 7B,C). Meanwhile, these microglia populations had the morphologic characteristics of enlarged somas with reduced and shortened processes (Figure 7D). Moreover, we applied FITC‐dextran combined with flow cytometry to accurately detect the phagocytosis of microglia. We found that FITC labeled microglia had a higher proportion which indicated that microglia were partially activated for enhanced phagocytosis (Figure 7E,F). Taken together, our results showed that a proportion of microglia were activated when the concentrate of lactate reached 12.10 mmol/L.

## 4 DISCUSSION

There were three major findings in the current study, which were collected in the following: 1) lactate accumulations and its concentrate significantly increased in both core and penumbra regions of ICH; 2) emodin has a good therapeutic effect on ICH rats; and 3) lactate accumulation was beneficial for the proliferation, cell survival, migration, and phagocytosis property of the microglia.

### 4.1 Lactate accumulation in hemorrhages

Metabolic changes have been observed in ICH model,^29^, ^30^ especially for lactate accumulation.^12^, ^30^, ^31^ There are several different approaches to the detection of lactate accumulation in former studies.^12^, ^26^, ^30^, ^31^, ^32^, ^33^, ^34^, ^35^ Lactate is a product of anaerobic glycolysis, and it is recognized as a reliable indicator of tissue ischemia. However, its concentration sharply changes after the animal dies.^34^ In order to avoid the influence of postmortem changes, several different methods have been developed, such as freezing anesthesia animal brain in situ with liquid nitrogen,^30^ freezing tissues in liquid nitrogen,^12^ microdialysis,^35^ and in vivo MRS method.^31^ Among these methods, the first three should be combined with analytical methods for the detection of lactate, such as spectrophotometric or enzymatic methods. Furthermore, the brain freezing method makes it difficult to obtain the tissues adjacent to the hematoma area; the tissue freezing method could not avoid the metabolic changes of post mortem; and the steps in the method of microdialysis are very complicated, despite having higher detection sensitivity. Thus in vivo MRS method should be the ideal approach for the detection of lactate directly, and is also suitable for clinical tests, along with undamaged MRI. In the spectrum of MRS, there are two separate proton signals for lactate (Figures 2 and 4). The height of the left one (~4.0 ppm) would be uncertainly influenced by the suppression of water signal. Therefore, it is not suited for quantitative analysis even though it was also significantly higher 3 days after surgery. The lactate signal in the higher magnetic field (~1.3 ppm) was used for quantitative analysis in this work despite mixing with lipid signal sometimes.

Lactate accumulation is a result of tissue ischemia and metabolic disorders of hemorrhages. After long‐term hemorrhages, the concentrations of other metabolites in the downstream of lactate should be altered, such as aspartate, glutamate, glutamine, and GABA. Due to the resolution of the in vivo NMR method, it was hard to detect the changes of glutamate, glutamine, and GABA directly. However, the aspartate's concentration did increase 3 days after surgery in the core region, and it was not detected in the penumbra region. This was consistent with the former finding in human beings^36^ and rats.^37^ Intracellular aspartate's increase was partially caused by the low oxygen availability and augmentation of the flux through the truncated TCA cycle.^38^

### 4.2 Potential role of lactate on recovering of hemorrhages

Lactate accumulation was associated with intracerebral hemorrhage, and it has been verified with the in vivo MRS method. However, there are only a few studies that discuss the function of lactate accumulation.^12^ Lactate accumulation has been verified as contributing to angiogenesis and neurogenesis in ICH model,^12^ which could interpret partial self‐healing after hemorrhages, especially in the penumbra area. Furthermore, we hypothesized that lactate accumulation could facilitate the migration and proliferation of the microglia cell. The lesion area of ICH has been divided to 4 regions (Figure 5A). The largest density of microglia/macrophages has been found in region III, which was close to the core region (region IV) of hemorrhages in our previous study. Thus, the proliferation or migration rate in this region should be much higher than the other areas. Furthermore, the morphologic analysis showed that the microglia moved from region I to region IV (core region).

We have found that the lactate concentration was gradually decreased from the core to the penumbra region, and the core area had the highest concentration. During the in vitro verification, the migration and proliferation rates of BV2 cell gradually increased following the increase of lactate concentration, but decreased after a certain value (12.10 mmol/L). Cell survival analysis showed that the relative survival rate of BV2 cell significantly decreased in the group of highest lactate concentration (24.20 mmol/L). However, there was no difference for the other groups, compared with the control group. Thus, the changes of the migration, proliferation, and survival rate were related with the lactate concentration, which was found with the in vivo MRS study in ICH animal model. Thus, the lactate accumulation in the brain should be an important factor for the movement and aggregation of microglia in the ICH animal model. Then, the aggregation microglia will help the angiogenesis and neurogenesis which was found in former studies.^12^

### 4.3 Lactate and medicine treatment

As a major effective constituent in the Traditional Chinese Medicine—Dachengqi decoction, rheum officinale (RO, Dahuang in Chinese) has been used to treat ICH for a long time.^39^, ^40^ The major active ingredients in RO are emodin, rhein, chrysophanol, physcion, aloe‐emodin, *etc* Among these chemicals, emodin has the highest concentration in the brain compared with the other chemicals, which is about 1/5 of the concentration in the blood. Thus, we assumed that emodin should be the major effective component in RO for the treatment of ICH diseases and was therefore also used to treat ICH in the animal model. Emodin could decrease the hemorrhage area, increase the recovery rate from ICH, and shorten the disease course, which was also shown based on the behavioral study.

The number of the microglia cell was decreased in the core region of the wounded area when it was higher than a certain value, which was also verified by in the in vitro analysis. The core region has the highest lactate concentration. After the treatment of emodin, the lactate concentration had the tendency to decrease in the core region of ICH. This might be good for the recovery of ICH. However, the lactate concentration in the penumbra region was increased after the treatment of emodin. The penumbra region had much lower lactate concentration, compared with the core region. Thus, a certain increase of the lactate concentration could also benefit from the recovery of the tissue from ICH. Taken together, the effect of emodin on the improvement of hemorrhage was comprehensive.

## 5 CONCLUSION

In the current study, we have demonstrated that lactate accumulation was always associated with the ICH symptom in the animals, and its concentration decreased from the core region to the penumbra area. The hemorrhagic area gradually decreased during the recovery of the intracerebral hemorrhage, especially when treated with emodin. Furthermore, the lactate concentration in the penumbra area also changed after the treatment of emodin. The in vitro culture study verified that lactate was beneficial for the phagocytosis property, proliferation, cell survival, and migration of the microglia cell. These data suggest that lactate plays quite an important role during the recovery of ICH, which could provide a novel therapeutic approach for ICH.

## CONFLICT OF INTEREST

The authors declare that there is no conflict of interest.

## Supporting information

Supplementary Material

## Data Availability

The datasets generated to obtain the results presented in this article are available from the corresponding authors on reasonable request (jie.wang@wipm.ac.cn).
