# Supplementary material for: Functions of lactate in the brain of rat with intracerebral hemorrhage evaluated with MRI/MRS and in vitro approaches
Source: CNS Neurosci Ther. 2020 Jun 2;26(10):1031–44. doi: 10.1111/cns.13399 (PMC7539841; doi:10.1111/cns.13399)
Supplement: Supplementary file 1 — Supplementary Material [file CNS-26-1031-s001.docx]

**Tables**

Table S1 Antibodies used in this study

| Antibody |  | Type | Dilution | Source |
| --- | --- | --- | --- | --- |
| CD68 |  | Mouse | 1:300 | Abcam |
| Iba-1 |  | Goat | 1:300 | Abcam |
| DAPI |  |  | 1:1000 | Abcam |
| DAM Alexa fluor 546  DAG Alexa fluor 488 |  | Mouse  Goat | 1:500  1:500 | Invitrogen  Invitrogen |


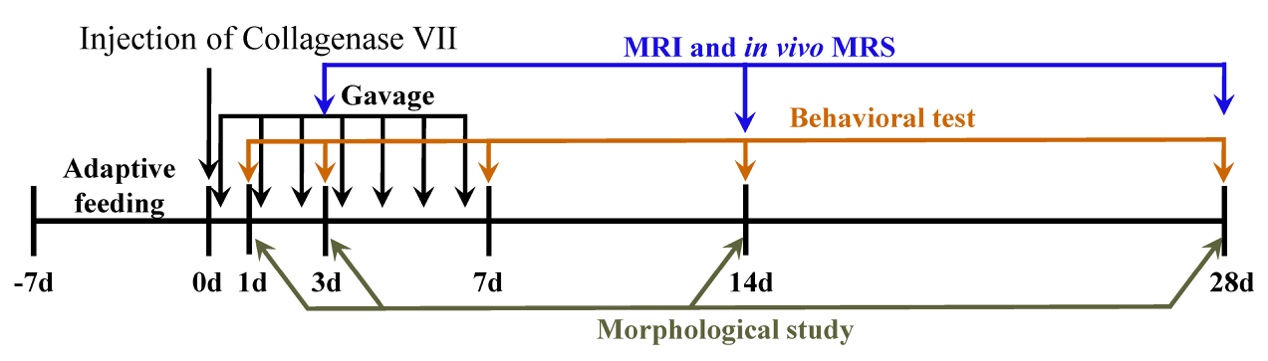
**Figure S1. The experiment flow chart.** The male SD rats (3 m-old) were adaptively bred for a week and then received left caudate putamen (CPu) infusion of 0.4 U collagenase VII (2 μL, ICH rats) or as a vehicle control, 0.9% NaCl (2 μL, sham rats). Afterwards, the rats received gavage with emodin or normal saline for 7 days after the CPu infusion 6 h later. Behavioristics were detected after the injection of 1 d, 3 d, 7 d and 14 d and 28 d and 6 rats were randomly selected from the groups to give the MRI test after the injection of 3 d, 14 d and 28 d.


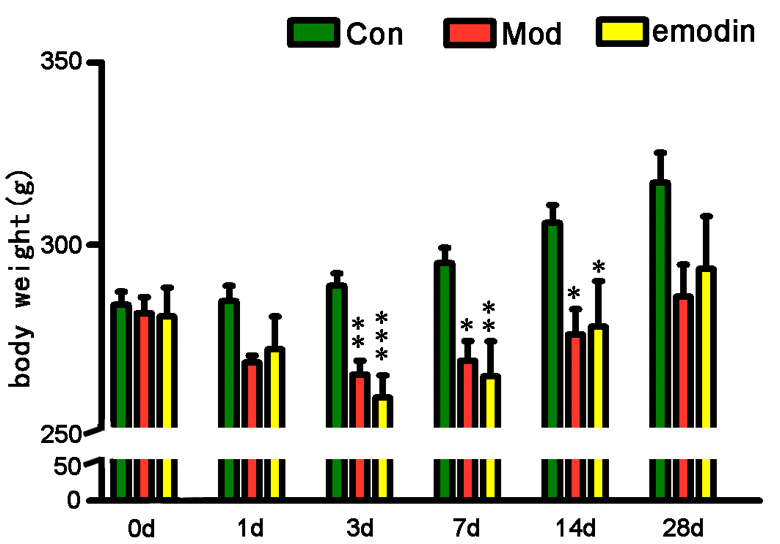


**Figure S2. The dynamic change of the rats’ weight.** The ICH model rats were treated with emodin and the dynamic change of weight were recorded after the injection of 1 d, 3 d, 7 d and 14 d and 28 d. The data were expressed as means ± SD (n=10). **p*<0.05，***p*<0.01，****p*<0.001 *vs* Con.
